# Limited reliability of the indirect immunofluorescence technique for the detection of anti-Rib-P antibodies

**DOI:** 10.1186/ar2548

**Published:** 2008-11-11

**Authors:** Michael Mahler, Jennifer T Ngo, Johannes Schulte-Pelkum, Tanja Luettich, Marvin J Fritzler

**Affiliations:** 1 Dr. Fooke Laboratorien GmbH, Mainstraße 85 D-41469, Neuss, Germany; 2 Department of Biochemistry and Molecular Biology, University of Calgary, 3330 Hospital Drive NW, Calgary, AB T2N 4N1, Canada; 3 Mikrogen GmbH, Floriansbogen 2-4 82061, Neuried, Germany

## Abstract

**Introduction:**

Autoantibodies to the ribosomal P proteins represent a highly specific marker for the diagnosis of systemic lupus erythematosus, where they have been associated with certain clinical manifestations. Historically, autoantibodies against ribosomal P proteins have been detected by indirect immunofluorescence, immunodiffusion, immunoblot, and other immunoassays. More recently, enzyme-linked immunosorbent assays and line and addressable laser bead immunoassays have become more widely used. The primary goal of this study was to determine the sensitivity of indirect immunofluorescence using conventional HEp-2 substrates in the detection of sera with ribosomal P antibodies as detected by other immunoassays.

**Methods:**

Anti-ribosomal P-positive sera (n = 345) as detected by an addressable laser bead immunoassay were collected between 2003 and 2007 and analysed by indirect immunofluorescence. Furthermore, 51 anti-ribosomal P-positive samples from an unselected systemic lupus erythematosus cohort (n = 100) and the Centers for Disease Control and Prevention (CDC) anti-nuclear antibody (ANA) reference sera were tested for anti-ribosomal P reactivity.

**Results:**

In the cohort of 345 anti-ribosomal P-positive samples identified by addressable laser bead immunoassay, a low sensitivity (<30%) of indirect immunofluorescence on HEp-2 cell substrates was observed. Although the degree of sensitivity varied among different manufacturers, all immunofluorescence substrates exhibited limited sensitivity and false-negative results were not restricted to samples with low anti-ribosomal P titers. Even the anti-ribosomal P reactivity of CDC ANA reference serum number 12 was not clearly predictable by indirect immunofluorescence. Comparison of five different methods for the detection of anti-ribosomal P found moderate qualitative agreements.

**Conclusions:**

Based on our data, we conclude that indirect immunofluorescence on HEp-2 cells is not a reliable screening test for the prediction of ribosomal P antibodies. As this method is widely used as a first-line screening test for anti-nuclear and other autoantibodies, special considerations for the detection of ribosomal P antibodies are needed. As with many other autoantibodies, further effort is required for the standardisation of ribosomal P immunoassays.

## Introduction

Although more than 25 years have passed since their first description as a highly specific biomarker for systemic lupus erythematosus (SLE) [1], autoantibodies (aab) to the ribosomal P proteins (referred to as Rib-P) have not achieved the attention or clinical utility that anti-Sm, anti-dsDNA (anti-double-stranded DNA), or anti-cardiolipin antibodies have. This might be attributed to the limited reliability of indirect immunofluorescence (IIF) assays for the detection of these aab, the lack of access to international reference serum samples, and the misunderstanding of their clinical relevance. The variation in the observed frequency of anti-Rib-P in SLE (approximately 10% to 40%) may be related to a number of factors but is largely dependent on patient selection and the test system used to detect the aab [2-4].

The Rib-P autoantigen consists of three protein components of the 60S ribosomal subunit which have been designated P0 (38 kDa), P1 (19 kDa), and P2 (17 kDa) [2]. A pentameric complex composed of one copy of P0 and two copies each of P1 and P2 interacts with the 28S rRNA molecule to form a GTPase domain, which is active during the elongation step of protein translation [2]. Historically, aab against these Rib-P and related antigens were detected by IIF [5], double immunodiffusion (DID), immunoblot (IB) [6-8], radioimmunoassay [9], and counter-immunoelectrophoresis. More recently, enzyme-linked immunosorbent assays (ELISAs) [3,10-14], line immunoassays (LIAs) [15], and addressable laser bead immunoassays (ALBIAs) [13] have achieved increasingly widespread use in clinical and research laboratories. Of note, several ELISA systems designed for research studies as well as clinical diagnostic applications have been produced and evaluated [3,7,12-14,16,17]. The Rib-P antigens used in these assays included purified native proteins, recombinant polypeptides, a synthetic peptide comprising the 22 C-terminal amino acids (C22), and a multiple-peptide construct [2,7,13,17,18]. Recently, two studies have shown that ELISAs with a mixture of the three Rib-P antigens yielded high sensitivity and specificity [3,14].

When human sera were tested by IIF on HEp-2 cell substrates, it was reported that anti-Rib-P antibodies produce a cytoplasmic staining pattern (CSP) that corresponded to the cellular location of the ribosomal P autoantigen [5]. Now that a variety of relatively sensitive techniques (that is, ELISA and ALBIA) are used in clinical laboratories, what is less well studied is the sensitivity or specificity of IIF as a screening test for the detection of Rib-P aab in relation to the sensitive confirmation assays. The objectives of this study were to analyse the sensitivity of IIF using conventional HEp-2 cells substrates for the detection of anti-Rib-P antibodies and to compare different state-of-the-art diagnostic technologies for the detection of anti-Rib-P antibodies.

## Materials and methods

### Sera

Three hundred forty-five serum samples that had a positive anti-Rib-P test as detected by an ALBIA (QuantaPlex ENA8; INOVA, San Diego, CA, USA) between 2003 and 2007 in the Mitogen Advanced Diagnostics Laboratory (Calgary, AB, Canada) were retrospectively analysed for aab by IIF on a HEp-2 substrate kit (HEp-2000; ImmunoConcepts, Sacramento, CA, USA) that included fluorescein-conjugated goat antibodies to human IgG (H+L). IIF patterns were read at serum dilutions of 1:160 and 1:640 on a Zeiss Axioskop 2 *plus *(Carl Zeiss, Jena, Germany) fitted with a 100-watt USHIO super-high-pressure mercury lamp (Ushio, Steinhöring, Germany) by two experienced technologists with more than 5 years of experience who had no knowledge of the ALBIA results.

Of the 345 anti-Rib-P-positive samples, 51 were randomly selected and tested for anti-Rib-P antibodies by ELISA (synthetic peptide; Dr. Fooke Laboratorien GmbH, Neuss, Germany), LIA (*recom*Line ENA/ANA IgG with recombinant P0; Mikrogen GmbH, Neuried, Germany), and EliA^® ^ribosomal P based on recombinant P0, P1, and P2 (Phadia, Freiburg, Germany), retested by IIF on HEp-2 cells, and analysed by IB as described by Mahler and colleagues [13], with some modifications (see below). Low (20 to 49 median fluorescence units, MFU), medium (50 to 100 MFU), and highly (>100 MFU) reactive samples (15 each) were selected based on the ALBIA results and analysed on HEp-2 slide kits from three different suppliers: INOVA, Euroimmun (Lübeck, Germany), and ImmunoConcepts. All tests were carried out according to the manufacturers' instructions for use. In addition, a clinically defined cohort of SLE patients (n = 100) who met the American College of Rheumatology (ACR) classification criteria [19] and the international Centers for Disease Control and Prevention (CDC) anti-nuclear antibody (ANA) reference sera [20,21] was assayed for anti-Rib-P antibodies by all methods. This study was approved by the Conjoint Biomedical Ethics Review Board at the University of Calgary.

### Immunoblot

An in-house IB with nuclear and cytoplasmic extracts from HeLa cells was employed for determination of Rib-P antibodies. In brief, nuclear and cytoplasmic extracts from HeLa cells were separated by SDS gel electrophoresis on a 13.5% polyacrylamide gel followed by transfer on nitrocellulose. After the nitrocellulose strips were blocked with casein hydrolysate buffer (CHB) (1% casein hydrolysate in phosphate-buffered saline [PBS]/Tween) for 30 minutes at room temperature (RT), they were incubated with serum samples diluted 1:100 in CHB for 1 hour at RT. After washings (3 × 5 minutes) with PBS/Tween, the strips were incubated 1 hour at RT with an alkaline phosphatase (AP)-conjugated anti-human IgG from Sigma-Aldrich (St. Louis, MO, USA) (A-3187), which was diluted 1:10,000 in CHB. The strips were washed again with PBS/Tween (3 × 5 minutes) and then equilibrated with AP buffer (pH 9.5) for 5 minutes and finally developed with the AP enzyme substrate NBT/BCIP (nitro blue tetrazolium/5-bromo-4-chloro-3-indolylphosphate). Development of the blots was stopped after 7 minutes by aspiration of the substrate followed by equilibration with distilled H_2_O. The IB strips were subjected to visual evaluation using a panel of reference strips, among them strips containing nuclear and cytoplasmic extracts that showed the characteristic Rib-P band pattern (37, 19, and 17 kDa). For evaluation of the blots, a subjective semi-quantitative scale was used: -, negative; (+), equivocal; +, weak positive; ++, moderate/strong positive; and +++, very strong positive. A serum was considered to be anti-Rib-P-positive when at least one of the three characteristic Rib-P bands was observed on both strips (nuclear and cytoplasmic).

### Statistical evaluation

The data were statistically evaluated using the Analyse-it software (version 1.62; Analyse-it Software, Ltd., Leeds, UK). Chi-square, Spearman correlation, and Cohen kappa agreement tests were carried out to analyse the agreement between portions, and *P *values of less than 0.05 were considered significant. Differences with *P *values of less than 0.05 were considered significant.

## Results

### Anti-Rib-P reactivity in approximately 20,000 consecutive samples

Over an audit period between 2003 and 2007 in a clinical diagnostic laboratory (Mitogen Advanced Diagnostics Laboratory), 345 of approximately 20,000 (approximately 2%) serum samples with anti-Rib-P reactivity were identified by ALBIA. Forty-five of these 345 samples (13%) were monospecific for anti-Rib-P antibodies in the context of other aab (chromatin, SS-A 60, Ro52, SS-B/La, Sm, U1-RNP, topoisomerase I, and Jo-1) detected by ALBIA. Only 35 of 345 (10.1%) of anti-Rib-P-positive sera displayed an IIF CSP consistent with the presence of anti-Rib-P antibodies as described in other studies [5]. The proportion of samples that had the typical IIF CSP increased when only moderate- and high-titer anti-Rib-P-positive samples were considered. Twenty-six of 145 (17.9%) moderate- and high-titer anti-Rib-P and 18 of 82 (22.0%) high-titer samples showed CSP. Eight of 345 (2.3%) anti-Rib-P-positive samples did not have any IIF staining pattern. Six of those samples had low titers and two high titers of anti-Rib-P antibodies by ALBIA.

### Confirmation of anti-Rib-P reactivity in 51 samples by other methods

When 51 samples with a positive anti-Rib-P test result by ALBIA were tested for anti-Rib-P reactivity by other assays, 13 of 51 (25.5%) samples were confirmed by LIA (recombinant P0), 27 of 51 (52.9%) by ELISA (synthetic peptide), 21 of 51 (39.6%) by EliA^® ^Rib-P, and 20 of 51 (39.2%) by IB. Of 20 samples with anti-Rib-P reactivity by IB, only 8 (40.0%) showed the IIF CSP that is considered to indicate the presence of anti-Rib-P antibodies (kappa = 0.19, *P *= 0.1514). The results of all methods showed no statistically significant agreement with an IIF CSP. When only medium and highly reactive (>1,000 MFU) samples as detected by ALBIA were considered, the percentage of confirmed results significantly increased (Table 1). The agreement between the individual methods and the IB was found at 0.57 (*P *< 0.0001) (ELISA), 0.71 (*P *< 0.0001), and 0.96 (*P *< 0.0001) according to the kappa method. Repeat analysis of these 51 sera by IIF on HEp-2 cells revealed CSP in 14 (27.5%) of the sera. The percentage of confirmed results did not increase significantly in medium- and/or high-titer samples for LIA and ELISA but did increase slightly for IIF.

**Table 1:** Sensitivity of LIA, ELISA, EliA^® ^Rib-P, IB, and IIF versus ALBIA

		All	Medium^a ^+ high MFU	Only high^b ^MFU
		n = 51	n = 27	n = 20
LIA	Positive + borderline, number (percentage)	19 (37.3)	9 (33.3)	7 (35.0)
	Positive, number (percentage)	13 (25.5)	8 (29.6)	7 (35.0)
ELISA	Positive + borderline, number (percentage)	36 (70.6)	21 (77.8)	14 (70.0)
	Positive, number (percentage)	27 (52.9)	17 (63.0)	12 (60.0)
EliA^®^	Positive + borderline, number (percentage)	22 (43.1)	12 (44.4)	8 (40.0)
	Positive, number (percentage)	21 (41.2)	11 (40.7)	8 (40.0)
IIF (CSP)	Positive, number (percentage)	14 (27.5)	11 (40.7)	8 (40.0)
IB	At least one Rib-P band, number (percentage)	20 (39.2)	10 (37.0)	7 (35.0)

^a^Medium 50 to 100 median fluorescence units (MFU); ^b^high ≥ 100 MFU. ALBIA, addressable laser bead immunoassay; CSP, cytoplasmic staining pattern; ELISA, enzyme-linked immunosorbent assay; IB, immunoblot; IIF, indirect immunofluorescence; LIA, line immunoassay; Rib-P, ribosomal P protein.

**Figure 1:**
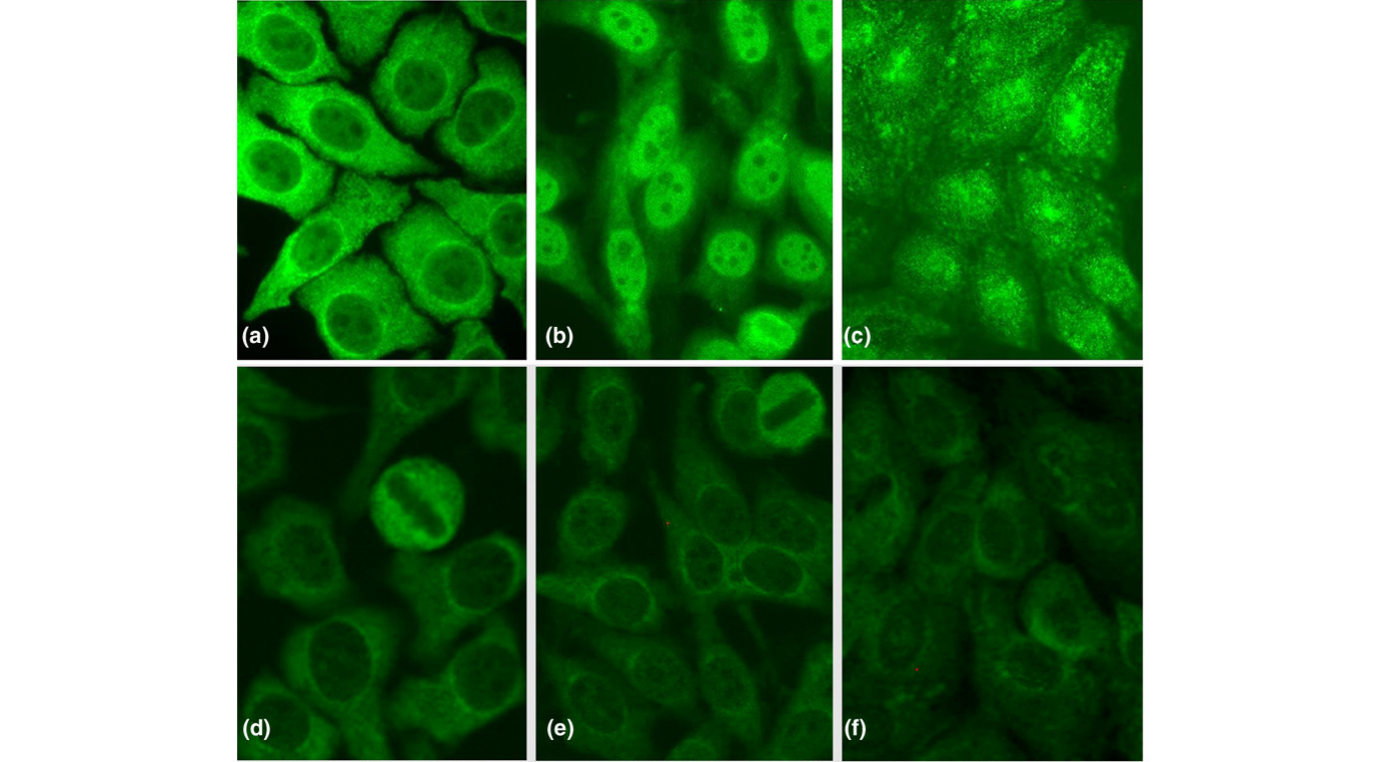
Indirect immunofluorescence staining pattern of anti-Rib-P-positive samples. One anti-Rib-P-positive serum that did not have autoantibodies to other known antigens **(a-c) **and the Centers for Disease Control and Prevention (CDC) anti-nuclear antibody reference serum number 12 **(d-f) **were tested at dilutions of 1:500 and 1:100, respectively, on slides from three different suppliers. Significant differences were observed in patterns of staining for the monospecific anti-Rib-P sera (a-c) on HEp-2 substrates from INOVA (San Diego, CA, USA) (a), ImmunoConcepts (Sacramento, CA, USA) (b), and Euroimmun (Lübeck, Germany) (c). Furthermore, the indirect immunofluorescence of the high-titer CDC anti-Rib-P reference serum produced only weak cytoplasmic staining on HEp-2 substrates from the same manufacturers (d-f). Rib-P, ribosomal P protein.

When a serum sample that was 'monospecific' for anti-Rib-P antibodies (no antibodies to dsDNA, Sm, U1-RNP, SS-A/Ro, and so on) was tested by IIF, it was noted that there was variation in the staining pattern produced on the HEp-2 substrates from three different manufacturers (Figure 1). We expanded this study to ensure that the limited sensitivity of IIF for the detection of anti-Rib-P antibodies is not restricted to slides of a certain manufacturer. Fifteen sera having low, medium, or high titers of anti-Rib-P as determined by ALBIA were analysed on HEp-2 slides from the three different suppliers (Table 2) and this analysis indicated two main observations. First, there is inter-manufacturer difference in the display of the typical CSP IIF patterns when medium or highly reactive sera were analysed and none of the manufacturers' HEp-2 substrates had a typical CSP pattern in sera with low levels of anti-Rib-P. Second, anti-Rib-P sera characterised by higher titer (MFU) tended to have a higher frequency of the typical CSP pattern, irrespective of manufacturer. Nevertheless, even on HEp-2 substrates from the manufacturer with the apparently highest frequency of CSP, less than 50% had the typical CSP Rib-P staining pattern.

**Table 2:** Presence of Rib-P-like cytoplasmic indirect immunofluorescence pattern on HEp-2 substrates from different manufacturers

Manufacturer	Low^a ^MFU, number (percentage)	Medium^b ^MFU, number (percentage)	High^c ^MFU, number (percentage)	All, number (percentage)
	n = 15	n = 15	n = 15	n = 45
INOVA (San Diego, CA, USA)	0	3 (20.0)	7 (46.6)	10 (22.2)
ImmunoConcepts (Sacramento, CA, USA)	0	1 (6.7)	5 (33.0)	6 (13.3)
Euroimmun (Lübeck, Germany)	0	0	3 (20.0)	3 (6.7)

^a^Low = 20 to 49 median fluorescence units (MFU); ^b^medium = 50 to 100 MFU; ^c^high ≥ 100 MFU. Rib-P, ribosomal P protein.

### Autoantibody profile of 51 anti-Rib-P-positive samples

To determine whether anti-Rib-P antibodies are commonly associated with other aab, the profiles of 51 anti-Rib-P-positive samples were established by LIA and ALBIA (Table 3). The highest prevalence of other concurrent aab in the Rib-P sera were anti-histone and anti-dsDNA by LIA and anti-chromatin by ALBIA, and the lowest prevalence was found for Jo-1 by LIA and for Jo-1 and SS-B by ALBIA. Anti-Rib-P antibodies were present in 33.3% of the dsDNA-negative sera and in 72.5% of the dsDNA-negative or borderline samples.

**Table 3:** Prevalence of autoantibodies in anti-Rib-P-positive samples (n = 51)

	Positive + borderline, number (percentage)	Positive, number (percentage)
Mikrogen ENA line assay^a^		
Histone	38 (74.5)	18 (35.3)
dsDNA	34 (66.7)	14 (27.5)
SmB	23 (45.1)	13 (25.5)
Ro52	22 (43.1)	17 (33.3)
SmD	21 (41.2)	6 (11.8)
SS-A/Ro60	21 (41.2)	13 (25.5)
Rib-P	19 (37.3)	13 (25.5)
RNPA	11 (21.6)	8 (15.7)
RNPC	8 (15.7)	5 (9.8)
SS-B/La	7 (13.7)	7 (13.7)
PCNA	6 (11.8)	3 (5.9)
RNP68	4 (7.8)	3 (5.9)
CENPB	3 (5.9)	2 (3.9)
Topoisomerase-I	2 (3.9)	1 (2.0)
Jo-1	1 (2.0)	1 (2.0)

ELISA		
Rib-P	36 (70.6)	27 (52.9)

ALBIA		
Rib-P	51 (100.0)	27 (52.9)
Chromatin	27 (52.9)	22 (43.1)
Ro52	13 (25.5)	12 (23.5)
SS-A/Ro60	11 (21.6)	8 (15.7)
RNP	8 (15.7)	2 (3.9)
Topoisomerase I	8 (15.7)	4 (7.8)
Sm	8 (15.7)	5 (9.8)
Jo-1	1 (2.0)	0 (0.0)
SS-B/La	1 (2.0)	1 (2.0)

^a^Mikrogen GmbH (Neuried, Germany). ALBIA, addressable laser bead immunoassay; ELISA, enzyme-linked immunosorbent assay; ENA, anti-extractable nuclear antigen; Rib-P, ribosomal P protein.

### Anti-Rib-P reactivity in a systemic lupus erythematosus cohort and controls

**Figure 2:**
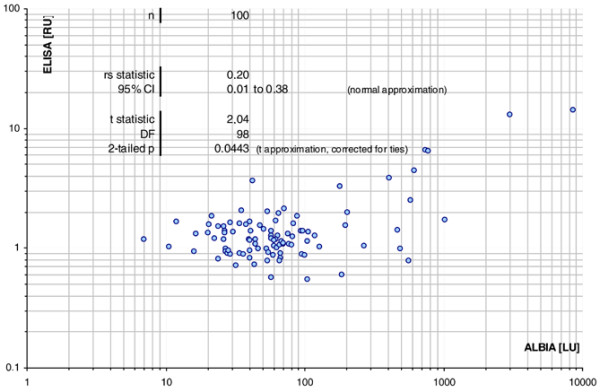
Correlation of addressable laser bead immunoassay (ALBIA) and enzyme-linked immunosorbent assay (ELISA). A correlation diagram was generated and the agreement was calculated according to Spearman, showing moderate agreement between the two assays. CI, confidence interval; DF, degrees of freedom; LU, luminescence units; RU, relative units.

Anti-Rib-P reactivity was then analysed in a cohort of 100 SLE patients by ALBIA, ELISA, IIF, and LIA. Sensitivity ranged from 11% (ALBIA) to 28% (ELISA), depending on the cutoff used. The quantitative agreement between the results of ALBIA and ELISA was 0.20 (confidence interval 0.01 to 0.38, two-tailed *P *< 0.0443) according to Spearman (Figure 2). The qualitative agreement between the different methods varied between 0.23 and 0.43 (kappa) (Table 4). To ensure that the high sensitivity of the ELISA was not accompanied by loss of specificity, we tested a panel of disease controls (n = 100), which showed 100% specificity. When anti-Rib-P reactivity was analysed in the context of anti-dsDNA antibodies as measured by LIA, it was observed that 4 of 27 (14.8%) dsDNA-negative SLE samples had anti-Rib-P reactivity by ALBIA, 3 (11.1%) by ELISA, and 1 (3.7%) by LIA. Four out of 66 (6.1%) dsDNA-negative or borderline samples were anti-Rib-P-positive by ALBIA, 11 of 66 (16.6%) by ELISA, and 5 of 66 (7.6%) by LIA.

**Table 4:** Agreement between different methods in a cohort of 100 patients with systemic lupus erythematosus

	ELISA (>1.5 RU) versus ALBIA (>350 LU)		
		
	ELISA +	ELISA -	
ALBIA +	8	3	11
ALBIA -	21	68	89
	29	71	
	ALBIA (>350 LU) versus LIA		
		
	LIA +	LIA -	
ALBIA +	5	6	11
ALBIA -	5	84	89
	10	90	

	ELISA (>1.5 RU) versus LIA		
		
	ELISA +	ELISA -	
LIA +	10	0	10
LIA -	19	71	90
	29	71	

	ELISA (>1.5 RU) versus IIF (CSP)		
		
	ELISA +	ELISA -	
CSP +	7	4	11
CSP -	22	67	89
	29	71	

	ALBIA (>350 LU) versus IIF (CSP)		
		
	ALBIA +	ALBIA -	
CSP +	3	8	11
CSP -	8	81	89
	11	89	

	LIA versus IIF (CSP)		
		
	LIA +	LIA -	
CSP +	4	7	11
CSP -	6	83	89
	10	90	

Kappa agreements			
	
	ALBIA (350 LU)	LIA	IIF
ELISA	0.29	0.43	0.23
LIA	0.38	-	-

ALBIA, addressable laser bead immunoassay; CSP, cytoplasmic staining pattern; ELISA, enzyme-linked immunosorbent assay; IIF, indirect immunofluorescence; LIA, line immunoassay; LU, luminescence units; RU, relative units.

### Anti-Rib-P reactivity in Centers for Disease Control and Prevention anti-nuclear antibody reference sera

Anti-Rib-P reactivity was determined in the sera of the CDC ANA reference serum panel by ELISA, LIA, ALBIA, and IIF on HEp-2 cells. When the CDC Rib-P reference (ANA #12) sample was tested at a dilution of 1:100 by IIF on commercial HEp-2 cell substrates, there was weak cytoplasmic staining on slides from all three manufacturers (Figure 1). However, when ALBIA, LIA, and ELISA methods of detecting anti-Rib-P antibodies were used, this reference serum showed strong reactivity (Table 5).

**Table 5:** Centers for Disease Control and Prevention reference standards tested for anti-Rib-P antibodies

Sample	Specificity	ELISA, RU^a^	Interpretation	ALBIA, LU^b^	Interpretation	LIA	Interpretation
CDC1	Anti-dsDNA	1.0	Borderline	143	Negative	0	Negative
CDC2	Anti-SS-B/La	0.3	Negative	97	Negative	0	Negative
CDC3	Speckled ANA	0.4	Negative	80	Negative	0	Negative
CDC4	U1-RNP	0.6	Negative	157	Negative	0	Negative
CDC5	Sm	0.6	Negative	89	Negative	0	Negative
CDC6	Nucleolar; anti-fibrillarin	0.3	Negative	210	Negative	0	Negative
CDC7	SS-A/Ro60	0.8	Negative	149	Negative	0	Negative
CDC8	Centromere	0.5	Negative	119	Negative	0	Negative
CDC9	Topoisomerase-I	0.9	Negative	166	Negative	0	Negative
CDC10	Jo-1	0.3	Negative	75	Negative	0	Negative
CDC11	PM/Scl	0.5	Negative	126	Negative	0	Negative
CDC12	Rib-P	6.6	Positive	11,664	Positive	3	Positive

^a^Cutoff = 1.5 relative units (RU); ^b^cutoff = 350 luminescence units (LU). ALBIA, addressable laser bead immunoassay; CDC, Centers for Disease Control and Prevention; dsDNA, double-stranded DNA; ELISA, enzyme-linked immunosorbent assay; LIA, line immunoassay; Rib-P, ribosomal P protein.

## Discussion

Although most studies have found that anti-Rib-P antibodies are highly specific for SLE, they have yet to achieve the clinical impact that anti-Sm or anti-dsDNA antibodies have [17,18]. The reported prevalence of anti-Rib-P aab in SLE ranges from 10% to 40% [2,3], a variation that may be related to several factors but appears to be significantly dependent on patient selection, the antigens used, and the diagnostic platform employed [2,3,11]. In a recent international multi-centre study of 947 SLE patients and 1,113 controls, the high disease specificity (99.3%) of anti-Rib-P antibodies was confirmed [3]. Of interest, in one patient initially diagnosed with rheumatoid arthritis, anti-Rib-P antibodies predicted the later clinical conversion into definite SLE [3,17]. Based on studies showing the high positive predictive value of anti-Rib-P, it has been proposed that, akin to anti-Sm and anti-dsDNA, anti-Rib-P may be considered for inclusion as a criterion for the classification of SLE [17,18].

The use of anti-Rib-P antibodies is often based on the understanding that they are a serological marker for neuropsychiatric SLE (NPSLE). Thus, in a clinical setting in which there is some doubt as to the etiology of neuropsychiatric events (for example, disease flare or pathogenesis), it is thought that the detection of anti-Rib-P can assist in the differentiation of NPSLE from psychoses and neurological features from other causes. Despite substantial investigation, the relationship between anti-Rib-P and organic central nervous system (CNS) involvement and/or NPSLE remains controversial [17,22-24]. A recent meta-analysis has shown low sensitivity and specificity and thus limited diagnostic value of anti-Rib-P for NPSLE [25]. These discrepancies have been attributed to methodological differences in aab detection, the difference in the criteria used to clinically define and identify various disease features, the demographics and/or the make-up of the patient cohorts, and the analysis of results [17].

Two recent investigations have provided new insights in the putative association between anti-Rib-P aab and CNS manifestations. Matus and colleagues [26] used human anti-Rib-P antibodies affinity-purified via an 11mer peptide derived from the C-terminal part of the cognate protein to demonstrate cross-reactivity with a new neuronal surface P antigen (NSPA). In that study, the affinity-purified anti-Rib-P antibodies caused rapid and sustained increase in calcium influx in neurons followed by apoptotic cell death. The epitope on NSPA responsible for the cross-reactivity has been speculated to consist of the Rib-P epitope core GFGLFD and acidic structures constituting a conformational epitope. In another recent investigation, anti-ribosomal P antibodies have been shown to be associated with CNS disease only at the time of diagnosis [27], an important observation that potentially explains previous controversies. In a mouse model, Katzav and colleagues [28] were able to induce depression-like behaviour via intracerebroventricular injection of affinity-purified anti-Rib-P antibodies. Thus, the detection of anti-Rib-P antibodies might be important not only for the diagnosis of SLE but also for the evaluation of CNS complications, particularly at disease onset and diagnosis.

### Difference in assay performance

The observed differences in sensitivity of the anti-Rib-P antibody assays in the present study are in agreement with findings from previous studies [2,7,13]. Apart from the methodological differences, two types of antigens were used in our study. The ELISA and ALBIA are based on a synthetic peptide (C22), whereas recombinant antigens were used in the LIA (P0) and EliA^® ^Rib-P (P0, P1, and P2). Although epitopes outside the major antigenic region (C22) are recognised by anti-Rib-P antibodies on ribosomal P0, the LIA had lower sensitivity when compared with other methods. This might be explained by the relatively higher epitope concentration of the C22 peptide in the ELISA and ALBIA.

### Limited sensitivity of indirect immunofluorescence and inter-manufacturer variation of patterns

The limited sensitivity of IIF for the detection of anti-Rib-P antibodies reported in this study might be attributed to a combination of different factors. These factors may include a limited antigen concentration in the cytoplasm, limited epitope exposure of the antigens in the 60S subunit of the ribosome, interference or masking by other aab that produce a variety of staining patterns, and a variety of cell preparation and fixation protocols used by different manufacturers. In one of the seminal studies of ribosome antibodies in SLE, it was noted that only 1% had the accompanying CSP on human organ sections [29]. This observation is concordant with our findings as approximately 10% to 20% of unselected SLE patients have anti-Rib-P antibodies and only a small proportion of these (approximately 10% to 30%) have a CSP on HEp-2 cells. Although Rib-P antibodies have been known in some detail for more than 20 years, it may have been that the limited sensitivity of IIF for the detection of anti-Rib-P antibodies was the reason these aab were not included in the classification criteria of SLE [30]. It is not surprising that higher frequencies of anti-Rib-P are now seen in SLE cohorts that correspond to the emergence of more sensitive assays such as ELISA and ALBIA [2,17]. Nevertheless, there is a prevailing notion that anti-Rib-P antibodies are commonly detected by IIF on HEp-2 substrates. Two case reports provide an interesting perspective on this topic [31,32]. Paller and colleagues [31] reported an 18-year-old, female, ANA-negative patient with anti-Rib-P antibodies that were detected by the relatively insensitive DID technique. In the second case report, published by Sugisaki and colleagues [32], a female ANA-negative patient with anti-phospholipid syndrome, lupus nephritis, and anti-Rib-P aab was described. Both reports emphasise the clinical importance of detecting anti-Rib-P antibodies in ANA-negative SLE patients.

Our data suggest that anti-Rib-P are not commonly manifest as an IIF CSP, although this was somewhat dependent on the manufacturer of the HEp-2 kits. The percentage of ALBIA Rib-P-positive sera that were reported to show a CSP was significantly higher in the small group of samples (n = 51) selected for further analysis compared with the routine cohort of 345 samples. In the initial cohort, staining patterns were read in the routine laboratory without special attention to the CSP. In contrast, the smaller cohort was retrospectively analysed with the focus on anti-Rib-P aab reactivity known to the investigator. In this context, it is important to mention that limitations in the sensitivity of the IIF test are not restricted to the detection of anti-Rib-P aab but have been observed for other aab (that is, SSA/Ro, SSB, and Jo1) [33-35]. In a previous study, the sensitivity of IIF as compared with an LIA as the primary assay for the detection of extractable nuclear antigen (ENA) antibodies was only 72% [33], and a significant portion of the IIF-negative/LIA-positive patients had clinical evidence of systemic autoimmune rheumatic disorders (SARD). Therefore, the authors concluded that, akin to IIF on HEp-2 cells, confirmation assays such as LIA should be performed when there is a clinical suspicion of SARD.

### Impact of anti-Rib-P antibodies on the diagnosis of systemic lupus erythematosus

Additionally, in a previous international study, 52 of 143 (36.3%) anti-Rib-P-positive samples have been reported as anti-dsDNA-negative [3]. This observation is in keeping with the findings of the present study, in which a significant portion of anti-Rib-P-positive samples show no anti-dsDNA reactivity. In patients who have anti-Rib-P antibodies but no antibodies to dsDNA or Sm, confirmatory serology may be missing and this in turn could result in an unfortunate delay of diagnosis and treatment of such patients. Accordingly, we recommend that the serological ACR criteria for the classification of SLE be reconsidered and revised to include anti-Rib-P.

## Conclusion

Based on our findings, we conclude that routine screening for aab by IIF on HEp-2 cell substrates has low sensitivity and, thus, limited reliability for the detection of anti-Rib-P in a routine clinical laboratory setting. Up to 90% of samples with anti-Rib-P reactivity, depending on the anti-Rib-P titer determined by ALBIA, were not accompanied by a CSP IIF staining pattern on commercially prepared HEp-2 cells. Although the difference between other anti-Rib-P assays (such as ELISA, IB, LIA, or EliA^®^) and the prevalence of IIF CSP was less pronounced, a significant portion of samples demonstrated no evidence of anti-Rib-P reactivity in IIF. Furthermore, we conclude that, as for many other aab, the standardisation of assays requires further effort and attention.

## Abbreviations

aab: autoantibodies; ACR: American College of Rheumatology; ALBIA: addressable laser bead immunoassay; ANA: anti-nuclear antibody; AP: alkaline phosphatase; C22: synthetic peptide comprising the 22 C-terminal amino acids; CDC: Centers for Disease Control and Prevention; CHB: casein hydrolysate buffer; CNS: central nervous system; CSP: cytoplasmic staining pattern; DID: double immunodiffusion; dsDNA: double-stranded DNA; ELISA: enzyme-linked immunosorbent assay; ENA: anti-extractable nuclear antigen; IB: immunoblot; IIF: indirect immunofluorescence; LIA: line immunoassay; MFU: median fluorescence units; NPSLE: neuropsychiatric systemic lupus erythematosus; NSPA: neuronal surface P antigen; PBS: phosphate-buffered saline; Rib-P: ribosomal P protein; RT: room temperature; SARD: systemic autoimmune rheumatic disorders; SLE: systemic lupus erythematosus.

## Competing interests

MM and JS-P are employed at Dr. Fooke Laboratorien GmbH, which sells the Rib-P ELISA. TL is an employee of Mikrogen GmbH, which manufactures diagnostic assays for autoimmune diseases. MJF is the director of Mitogen Advanced Diagnostics Laboratory, which provides diagnostic testing services. JN declares that she has no competing interests.

## Authors' contributions

MM developed the Rib-P ELISA, contributed to the study design, evaluated the data, and participated in the preparation of the manuscript. MJF contributed to the study design, provided laboratory resources, and participated in the preparation and revisions of the manuscript. JS-P participated in data analysis and the preparation of the manuscript. TL helped in the evaluation of data. JTN performed Rib-P assays and contributed to the data analysis. All authors read and approved the final manuscript.
